# Mother–Pup Interactions: Rodents and Humans

**DOI:** 10.3389/fendo.2014.00017

**Published:** 2014-02-26

**Authors:** Aldo B. Lucion, Maria Cátira Bortolini

**Affiliations:** ^1^Departamento de Fisiologia, Instituto de Ciências Básicas da Saúde, Universidade Federal do Rio Grande do Sul, Porto Alegre, Brazil; ^2^Departamento de Genética, Instituto de Biociências, Universidade Federal do Rio Grande do Sul, Porto Alegre, Brazil

**Keywords:** mother–infant attachment, oxytocin receptor gene, innate behavior, social behaviors, behavior synchrony

## Abstract

In order to survive after birth, mammalian infants need a caretaker, usually the mother. Several behavioral strategies have evolved to guarantee the transition from a period of intense caregiving to offspring independence. Here, we examine a selection of literature on the genetic, epigenetic, physiological, and behavioral factors relating to development and mother–infant interactions. We intend to show the utility of comparisons between rodent and human models for deepening knowledge regarding this key relationship. Particular attention is paid to the following factors: the distinct developmental stages of the mother–pup relationship as relating to behavior; examples of key genetic components of mammalian mother–infant interactions, specifically those coding for the hormones oxytocin and vasopressin; and the possible functions of gene imprinting in mediating interactions between genetics and environment in the mother–infant relationship. As early mother–infant attachment seems to establish the basic parameters for later social interactions, ongoing investigations in this area are essential. We propose the importance of interdisciplinary collaboration in order to better understand the network of genes, gene regulation, neuropeptide action, physiological processes, and feedback loops essential to understand the complex behaviors of mother–infant interaction.

## Introduction

A social interaction is a dynamic process, composed not of a single set of behaviors at a given moment, but rather of the relationship of a series of behaviors over time. The exchange of information between subjects progressively modifies them. Due to its fundamental role in development, the effects of mother–infant interaction last throughout life.

In this review, we examine research on behavioral strategies that have evolved to guarantee the transition from intense caregiving to offspring independence. Infants display preference for social stimuli, suggesting evolutionary pressure in favor of these stimuli. In humans, the mechanisms involved in mother–infant attachment are still insufficiently understood. Nevertheless, a few areas of research are offering promising insight into the determination of this complex interaction. First, we examine studies that demonstrate olfactory stimuli from the mother to be crucial for infant latching and suckling, a determining factor in mother–infant attachment, feeding, and infant survival (1). Next we offer case studies on the genetic basis of two neurotransmitters, oxytocin (OXT) and vasopressin, which facilitate social recognition by acting on several brain regions, such as the olfactory bulb. In placental mammalians, both OXT and vasopressin are highly involved in key social interactions, including social recognition, pair bonding, and parental behavior. Finally, we consider the possible role of imprinting of candidate genes in determining features of the mother–infant interaction. These fundamental early-life physiological, genetic, and epigenetic processes suggest why, in many ways, mother–infant attachment establishes basic parameters for later social interactions.

## The Actors: Mother and Infant

In rodents and humans, mother–pup interaction involves two essential types of actors: the mother and the infant, or several infants at the same time. Many other elements may be involved and/or affect this interaction: the father, the environment, siblings, and predators (2). However, here we focus on mother and infant. This particular relationship involves two organisms in different developmental stages. In this sense, it is an asymmetric relationship.

Moreover, this interaction occurs over time and is constantly adjusted, especially due to the fact that one organism – the infant – changes dramatically over the course of the relationship. Motherhood also involves behavioral changes, such as long-term memory of mothering mediated by OXT (3) and selectively reduced stress responses (4, 5) and anxiety (6).

Animal models have been developed to analyze the effects of maternal deprivation on the development of offspring (1, 7). Due to the complexity of the neuroendocrine and behavioral peculiarities of this relationship (8), the alteration of one element can induce changes of several orders, many of them not directly related to the original disturbance. Two changing organisms interact in a complex and balanced dynamic (9). Attempts to replace a missing mother or to repair an affection poor childhood (10) are rather difficult. This difficulty probably depends on the fact that there are at least two organisms that are necessarily changed by the interaction.

A mother–infant interaction is unique and depends on many factors (11). For instance, even in large litters, as in rats, mothers show different maternal behaviors toward male and female offspring (12). There are several elements that should be taken into consideration: the genetic background of the mother and the infant; the past experiences of the mother and the prenatal environment of the infant; the neuroendocrine profile of mother and infant; the stress copying style of the mother; and the environment (social and resource availability), among others. To consider such a wide variety of factors is a challenging task. As such, it is essential for the neuroscientist to collaborate with geneticists and physiologists in order to understand the origins of relevant psychopathologies (13).

## Time: Lifecycles and the Development of the Mother–Infant Interaction

The mother–infant interaction depends upon several elements to establish attachment: the product of genes such as hormones and/or neurotransmitters (e.g., OXT, vasopressin, serotonin, noradrenaline), cytokines (14), opioids (15, 16), and dopamine (17), as well as epigenetic (18) and environmental factors (19). In mammals, the uterus is the first site of mother–infant interaction (20, 21). In both rodents and humans, pregnant females undergo physiological and behavioral changes to support gestation (22); and several studies have shown that the infant responds to the mother from within the uterus (23, 24). During the neonatal period, mother–infant attachment develops and filial bonds form (25, 26). This period seems crucial for behavioral development and the establishment of the infant’s stress coping style (27). The mother’s behavior conveys important information about the environment in which the newborn will live (28), through sensory stimulation of pups by the mother (29). Additionally, the mother–infant interaction involves the behavior of the mother in synchrony with that of the infant (30). Predictability of mother’s behavior and thus a secure attachment is crucial for the neurocognitive development of offspring (31).

Consistent demonstrations in rats have shown that mother–infant interaction is based on infant learning processes, expressed by increased CREB phosphorylation (19) and the development of a memory of the mother (4). This is a simple mechanism involving the noradrenergic activity of the locus coeruleus on the olfactory bulb, where the memory of the mother begins (32). Two aspects seem important: the physiology of the locus coeruleus, whose activity is less inhibited by its noradrenergic inhibitory autoreceptors during infancy than later in life; and plastic changes induced by the learning process. Environmental stimuli (odor and varying forms of body contact with the mother) act on the neural system of the infant, which is particularly receptive and prepared to react to those stimuli; the system has a very low threshold during this specific period. All of these interactions exert long-lasting impact on the development of the infant.

## Origins of the Mother–Infant Interaction: Nature versus Nurture

The innateness of behaviors is controversial. Some authors suggest that behaviors presented by an individual early in life are essentially based on the genome and are innate (2, 33, 34). The behaviors would be already “wired” and triggered by specific stimuli, during sensitive period (35). However, consistent studies have shown that mother–infant interaction involves learning mechanisms (4, 11, 36). Physical contact is crucial to establish bonding. In rats, the mother’s licking induces olfactory memory that would be the basic mechanism of the mother–infant interaction. This interaction is by its nature dynamic and induces permanent reorganizations of the neuroendocrine systems and behaviors of both mother and offspring. Moreover, the system is open to the environment and can be affected by environmental stimuli in several ways, inducing short-term and/or long-term changes (37).

For the mother–infant interaction, it seems plasticity is not exclusively related to specific learning periods, as in other functions. For comparison, we might look at the classic neurophysiological example of ocular dominance columns in the visual system (38). These columns exist in rudimentary form at birth, but need to be stimulated by specific stimuli during a set period to fully develop their function. In the case of the mother–infant interaction, we would predict that plasticity is not time-limited as in the development of ocular dominance columns, but rather is cumulative. The constant learning processes that occur over time in the mother–infant relationship provoke related plastic changes. Thus the development of this interaction and the consequent attachment would not be an all-or-nothing phenomenon. Nevertheless, there are periods of greater impact, such as birth and the initial contact directly following birth.

In order to conceptualize the origins of the mother–infant interaction, let us consider an analogy between that interaction and the rhythmic activity of locomotion. Although these two behaviors are very different in complexity, it is interesting to imagine a similar basic working model. In this model, for locomotion, the output of a hierarchical neural system is the result of the activity of several circuits working in coordination. This system could be triggered by specific environmental stimuli. The generation and fine control of locomotion involves a basic central pattern generator in the spinal cord modulated by several other hierarchical disposed structures (39). In the case of the mother–infant interaction, this model would imply that several “layers” of interconnected neuroendocrine structures are the basis for behavior. The central pattern generator would first need to be properly triggered and then modulated by the interaction that it generates. Feedback is essential in social behaviors; the interaction generated by a behavior can be modulated by the response of the partner. However, in its very early stage, a supposed innate “central pattern generator” would require feed-forward action. Based on this model of the development of the mother–infant interaction, the functional characteristics of the infant locus coeruleus during the period after birth would be an innate system, prepared to be stimulated by the mother.

Animals are born with the capacity to develop complex behaviors (40), but not with behaviors *per se*: an infant must build them. The constructive nature of this process is analogous to those of other systems; for instance, in cognitive functions, an animal builds internally a unique sensation of a particular stimulus.

## Role of Genes

It is well known that behavioral traits such as parent–infant bonding are the product of a sophisticated combination of genetic, environmental, and epigenetic factors. The adequate interplay of these elements, both over the course of the evolutionary trajectory of a species, and during the development and life of an individual, ensures a behavioral repertoire sufficient for survival and reproductive success. Identifying the contribution of genetic factors is the starting point for understanding the formation and expression of a particular phenotype. Although there is still much to learn regarding the genes implicated in specific mammalian behavioral phenotypes, a number of them have been characterized. This is the case of the genes for the OXT and arginine vasopressin (AVP) nonapeptides, which act as hormones and neurotransmitters.

It has been proposed that *OXT* and *AVP*-like genes originated due to a tandem duplication of an ancestral gene ~500 million years. This event would explain the presence of at least one homolog in jawed vertebrates (41), although many aspects of the origin and evolution of these genes remain unknown (42). In humans, *OXT* and *AVP* are located on the same chromosome, at 20p13, but with opposite orientation (43).

In placental mammalians, OXT and AVP nonapeptides differ by two amino acids at positions 3 and 8 (OXT: Cys–Tyr–Ile–Gln–Asn–Cys–Pro–Leu–Gly, and AVP: Cys–Tyr–Phe–Gln–Asn–Cys–Pro–Arg–Gly) and play an important role during lactation, uterine contractions, offspring care, stress-response, and other reproductive and social behavioral traits (41, 44, 45).

Although they boast a wide spectrum of activity, OXT and AVP are best known for their contribution to the regulation of social behaviors. Pedersen and Prange (46) were pioneers in showing that artificial administration of OXT promoted maternal nurturing in virgin and steroid-primed female rats, indicating that this hormone plays a specific role in the mother–pup interaction. Other authors replicated and extended this initial study in several other mammalian species, including humans [(45) and references therein]. For example, Bosch (47) showed that the OXT and AVP systems are key factors regulating maternal aggression and anxiety in rodents, while Apter-Levi et al. (48) found that both OXT and AVP are associated with human maternal and paternal behaviors in distinct, sometimes overlapping, ways.

Both nonapeptides are highly conserved in placental mammalians, with a few rare exceptions. For example, Lee et al. (49) showed that five New World monkey species had fixed a T > C mutation changing leucine to proline at position 8 of OXT. Other authors described an *AVP* mutation leading to a substitution of arginine for lysine at position 8 in opossums and pigs, but the phenotypic consequences of these changes remain unknown (42, 50). It is noteworthy that changes at amino acid chain position 8 characterize some non-placental AVP/OXT-like nonapeptides such as mesotocin in marsupials (51, 52).

The oxytocin receptor (OXTR) and the three AVP receptors (A2, V1a, and V1b) are encoded by the *OXTR, AVPA2, AVPR1a*, and *AVPR1b* genes located on human chromosomes 3, X, 12, and 1, respectively. These receptors, which use G-proteins as transducer signals across the cell membranes, have seven transmembrane domains, four extracellular, and four intracellular domains. Both OXT and AVP can bind to each one of the four above-indicated receptors, but not with the same affinity, since the most important binding sites differ slightly among the four receptors (53, 54).

Oxytocin and AVP are produced in greatest quantity in the hypothalamus, but their activity outside of the brain depends on their interaction with the receptors produced in various organs and tissues. For instance, the presence of OXTR in the uterus and mammary glands guarantee uterine contraction and milk ejection (42, 55). Hammock and Levitt (56), on the other hand, showed that OXTR is also found in several tissues of the mouse embryo, such as the adrenal glands, brown adipose tissue, and the oronasal cavity.

In contrast to the observed *OXT* and *AVP* gene conservation, hundreds of mutations in the *OXTR, AVPR2, AVPR1a*, and *AVPR1b* genes have been reported in placental mammalians. For instance, in pigs, *AVPR1a* and *AVPR1b* mutations have been associated with stress and aggressive behavior through their connection with ACTH, an important component of the hypothalamic–pituitary–adrenal (HPA) axis (57, 58). In humans, polymorphisms in these receptor genes have also been linked to attachment, generosity, and pair bonding behaviors (59).

These examples illustrate that variations in candidate genes can be related to some intra and inter-specific mammalian behaviors. As seen above, however, other non-genetic factors have important roles in determining behavioral phenotype; this may be the case for the differential care of offspring between father and mother. Although males and females can have the same alleles in genes related to pup-care behaviors, only ~10% of mammalian species show significant male parental care, the majority of which are primates, carnivores, and rodents (60). This suggests that some social behavior candidate genes, located on autosomal chromosomes, can be subject to the imprinting process, an epigenetic phenomenon where changes in gene function can be heritable despite the fact that no alteration in the DNA sequence is detected. If genetic variation in key peptides and their receptors is understood as a first step, the imprinting of genes can be understood as a second step in determining the features of the mother–infant interaction. Below, we will see some examples of imprinted genes.

## Imprinted Genes

The term “imprinted genes” refers to genes in which either the maternal or paternal copy is exclusively expressed. Previous study (61) presented evidence that the imprinted gene *PEG3* is involved in sexual behavior and it would also be related to maternal care. More recently, Garfield et al. (62) showed that the imprinted gene *GRB10* influences, in mice, distinct physiological processes, fetal growth, and adult social behavior, due to actions of the two parental alleles in different tissues. An evolutionary explanation for this parent-of-origin-dependent gene expression is the “conflict hypothesis.” A good example of evidence for this hypothesis, which will also help illustrate the meaning of imprinted genes, is the case of insulin-like growth factor (IGF2) and its receptor (IGF2R). As it is an imprinted gene, organisms that predominantly express the paternal allele of *IGF2* show high levels of offspring resource extraction from the mother. However, if selection favors high expression of the maternally inherited allele of *IGF2R*, the offspring would show low levels of resource extraction from the mother (63). Thus, the balance of these imprinted genes would define a very initial and essential relationship pattern between mother and offspring interaction regarding nurturing.

Nevertheless, the likelihood of epigenetic influence on imprinted genes raises the question of the importance of environmental intervention in determining behavior. In rats and humans, several studies have shown that changes in the expression of candidate genes due to epigenetic mechanisms are connected with early parental care. Furthermore, McGowan et al. (64) suggested that the epigenetic response to maternal care in rats does not involve a single candidate gene, but rather includes changes in the expression of hundreds of additional genes. It is possible to speculate that the same happens with other placental mammalians, including humans.

In conclusion, early-life interactions clearly mold the neural basis for social behaviors (65), and sociability is perhaps the most important natural evolutionary force for mammalian species (66). In this context, it can be assumed that interactions with the mother are essential, not only in quantity, but perhaps most importantly in quality. A crucial question that emerges, among many, is whether it is possible to predict the consequences of the absence of a mother for her offspring, since several studies have indicated a genetic basis for animal behavior.

## Conflict of Interest Statement

The authors declare that the research was conducted in the absence of any commercial or financial relationships that could be construed as a potential conflict of interest.
